# Impact of potentially inappropriate psychotropic medicines on falls among older adults in 23 residential aged care facilities in Australia: a retrospective longitudinal cohort study

**DOI:** 10.1136/bmjopen-2024-096187

**Published:** 2025-04-09

**Authors:** Narjis Batool, Magdalena Z Raban, Karla Seaman, Johanna Westbrook, Nasir Wabe

**Affiliations:** 1 Centre for Health Systems and Safety Research, Australian Institute of Health Innovation, Macquarie University, Sydney, New South Wales, Australia

**Keywords:** Aging, Medicine, Nursing Homes

## Abstract

**Abstract:**

**Objective:**

Falling is common among older adults in residential aged care facilities (RACFs) and potential inappropriate psychotropic medicines (PIPMs) use may increase the risk of falling. This study aimed to determine the impact of PIPMs on falls using longitudinal observational data.

**Methods:**

A retrospective longitudinal cohort study was conducted using routinely collected electronic health data from 23 RACFs in Sydney, New South Wales, Australia. The study included 3064 permanent residents aged ≥65 (2020–2021). PIPMs were identified using updated Beers criteria 2023. We considered three fall outcome groups: all falls, injurious falls and falls requiring hospitalisation. The falls incidence rates (IRs) were calculated for overall residents in RACFs as well as for central nervous system (CNS)-PIPM users and non-users. We applied a zero-inflated negative binomial regression model to assess the association between falls and CNS-PIPMs.

**Results:**

A total of 40% (n=1224) of long-term care residents used at least one CNS-PIPM and 10% of residents (n=302) used two or more. The falls IRs of CNS-PIPM users were 16.2 falls per 1000 resident days (95% CI 15.9 to 16.5) for all falls, 5.68 falls per 1000 resident days (95% CI 5.48 to 5.88) for injurious falls and 1.77 falls per 1000 resident days (95% CI 1.66 to 1.88) for falls requiring hospitalisation, whereas the falls IRs of non-CNS-PIPM users were 10.8 falls per 1000 resident days (95% CI 10.6 to 11.0) for all falls, 3.65 falls per 1000 resident days (95% CI 3.52 to 3.78) for injurious falls and 1.26 falls per 1000 resident days (95% CI 1.19 to 1.33) for falls requiring hospitalisation. CNS-PIPM users had a significantly greater rate of falls overall compared with non-users (IRR 1.29; 95% CI 1.16 to 1.44) for all outcomes.

**Conclusions:**

Falls are frequent among CNS-PIPM users resulting in injury and hospitalisation, with 70% of CNS-PIPM users falling at least once and one in three requiring admissions to hospital. Reviewing residents’ use of psychotropic medicines should be considered as part of strategies to reduce falls incidence among older adults in RACFs.

**STRENGTHS AND LIMITATIONS OF THIS STUDY**

- The study’s strength lies in its multicentre design and the use of a comprehensive measure of falls (three types of falls) among central nervous system psychotropic medicine users and non-users.
- This study used data about administered medicines rather than prescribed medicines.
- A limitation of the study is that the results focus on residential aged care facilities (RACFs) from one provider and therefore may not be representative of all RACFs in Australia.
- Furthermore, we evaluated the medicines flagged as potentially inappropriate in the Beers criteria 2023, but we did not have detailed clinical information about each resident and therefore could not confirm whether they were indeed misused or actually inappropriate.

## Introduction

 Falls are a significant public health concern globally, with their clinical consequences including injuries, psychological distress, disability, economic burden, poor quality of life and even death.^1^,^3^ According to the WHO, falls are the second dominant cause of injury deaths, with an approximate death rate of 684 000 individuals annually, and are accountable for over 38 million disability-adjusted life years lost every year worldwide.^4^ Falls are a common event among older adults, with more than 25% of those over 65 years in the community^5^ and over 50% of those living in residential aged care facilities (RACFs) (also called care homes, nursing homes or long-term care) falls annually.^6^ In Australia, falls are responsible for 43% of injury hospitalisation and 42% of injury death among older adults.^7^ Older adults in Australian RACFs experience six times more fall-related injuries as compared with older adults of the same age in the community.^8^ A study among 6655 residents from 25 RACFs reported that there were 7.14 falls per 1000 resident days.^9^ Older adults in RACFs are more likely to fall due to advanced age, multimorbidity, cognitive impairment, fall history and poor family and social support.^10^

Falls can result from extrinsic (eg, slippery surfaces, poor lighting, unsafe footwear) and/or intrinsic factors (eg, medicine use).^11 12^ Older adults often experience mental health conditions such as anxiety, sleep disturbance, depression and dementia,^13^ which are commonly treated by psychotropic medicines.^14^ Psychotropic medicines are considered to increase the risk of falls by inducing cognitive and psychomotor slowing through their direct effect on the central nervous system (CNS).^15^ Psychotropic use is high and often inappropriate among residents of RACFs globally.^16^ A study among 2555 residents from 27 nursing homes in Italy found that 63.2% of residents used at least one potentially inappropriate psychotropic medicine (PIPM).^17^ Another study of 5825 older adults with dementia from 68 RACFs in Australia reported that 65% of residents had used the antipsychotics for significantly longer than the recommended targeted 3 months with the annual use ranging between 27.6% and 32.6% over a 4-year period.^18^ Inappropriate medicine use has been associated with adverse health outcomes such as falls.^19^

In Australia, studies have investigated the association of falls with the use of falls risk increasing drugs (FRIDs) in the community and RACFs,^20^,^22^ but these were mostly cross-sectional, retrospective studies with small sample sizes, short follow-up periods and data from single centres. A recent longitudinal study was conducted in RACFs to examine the FRID association with falls but did not investigate potentially inappropriate FRIDs, excluded anti-Parkinson’s drugs and did not consider falls requiring hospitalisation.^23^

Using contemporaneous data on medications administered to residents, we aimed to conduct a retrospective, longitudinal cohort study to determine the impact of PIPMs on all falls, injurious falls and falls requiring hospitalisation, in RACFs in Sydney, NSW, Australia. The purpose was to generate new evidence to understand the contribution of PIPMs and falls among older adults to inform the design of fall prevention programmes in RACFs.

## Methods

### Study design and setting

A longitudinal cohort study was conducted using routinely collected de-identified electronic health record (EHR) data from 23 RACFs in Sydney, Australia.

### Participants

All permanent residents aged ≥65 who were present in the RACFs at any time from 1 January 2020 to 31 December 2021 were included. Non-permanent residents (interim or respite care residents) were excluded.

### Data source

The EHR data were extracted from three systems: residential profile data, medication administration data and incident data. The residential profile data have information related to a resident’s demographic characteristics (eg, age, gender, date of admission, date of departure). Medication administration data contain details of each medicine administered (eg, medicine names and time of administration). The incident reports contain information about fall incidents (eg, falls location and types). Medicines were mapped to the WHO’s Anatomical Therapeutic Chemical (ATC) classification system.^24^

### Potentially inappropriate psychotropic medicine exposure

PIPMs were identified according to updated Beers criteria 2023.^25^ Beers criteria are widely used globally to identify potentially inappropriate medicines in older adults and include criteria for use of psychotropic medicines. The criteria were first published in 1991, and the latest version of the criteria was updated in 2023. Beers criteria 2023 have five categories. We used only the first category, which includes potentially inappropriate medicines in older adults. Medicines listed under the CNS drug class from the first category were included in this study and identified using ATC codes (online supplemental table 1). The CNS drug class includes antidepressants with strong anticholinergic activity, antiparkinsonian agents with strong anticholinergic activity, antipsychotics first- (typical) and second- (atypical) generation, benzodiazepines, barbiturates and non-benzodiazepine receptor agonist hypnotics (‘Z-drugs’).

Our decision to focus specifically on CNS drugs was guided by the availability of clinical information within our dataset. Many drug classes included in the first category of the Beers criteria require additional clinical details such as laboratory values, diagnostic history and comorbid conditions that were not comprehensively captured in our dataset. In contrast, CNS drugs could be assessed with the available data without the need for extensive additional clinical context. Given the strong evidence linking CNS medications to fall risk, we prioritised evaluating this group to ensure robust and meaningful findings within the constraints of our dataset.

We used the updated Beers criteria 2023 because it is not disease-specific and requires less clinical information regarding a disease under the CNS category, making it more suitable for our dataset where limited clinical details were available. The addition, deletion and revision of certain potentially inappropriate medicines in updated Beer criteria 2023 enhanced its applicability in our study.

The significant changes made in the updated Beers criteria 2023 for CNS medicines are: (1) removal of several CNS medicines from the first category of Beers criteria 2023, that is, protriptyline, trimipramine, amobarbital, butobarbital, mephobarbital, pentobarbital, secobarbital, flurazepam and quazepam. The potentially inappropriate CNS medicines were removed due to their low utilisation or no longer available in the USA, (2) the inclusion of potentially inappropriate anti-Parkinson’s drugs in Beers criteria 2023, which were absent in Beers criteria 2019 and (3) the new version 2023 has modified and clarified some statements; for example, it is clarified that the antidepressant criteria refer to antidepressants with strong anticholinergic activity.

In the Beers criteria 2023, potentially inappropriate antipsychotic medicines under the CNS drug class have clinical recommendations, that is, ‘Avoid, except in FDA-approved indications such as schizophrenia, bipolar disorder, Parkinson disease psychosis, adjunctive treatment of major depressive disorder, or for short-term use as an antiemetic’. This clinical information for antipsychotic medicines was applied to indications such as schizophrenia and bipolar disorders due to our data limitations. Residents who were using at least one potentially inappropriate CNS medicine were classified as a CNS-PIPM user.

### Outcome measures

We considered three fall outcomes: all falls, injurious falls and falls requiring hospitalisation. All falls were defined as any falls event that occurred regardless of any body injury or requiring hospitalisation. Injurious falls were those that resulted in any type of body injury (eg, hip injury, head injury). Falls requiring hospitalisation were those that required admission to hospital for further assessment or management.

### Patient and public involvement

None. The ethics committee granted us a waiver of consent since the study used de-identified retrospective data, and obtaining consent from the participants was deemed impracticable.

### Statistical methods

We reported the falls incidence rate (IR) per 1000 resident days for overall residents in RACFs as well as for CNS-PIPM users and non-users. IRs were calculated by dividing the number of falls incidents by the number of resident days. We conducted a zero-inflated negative binomial (ZINB) regression model to compare the difference in the incidence of falls by CNS-PIPM users and non-users. We controlled for age, sex and health status (ie, arthritis, fracture, dementia cognitive impairment, anxiety, diabetes mellitus, visual impairment, delirium, Parkinson’s disease and ATC level 1 medicines), as these factors have been demonstrated in the literature as falls risk factors.^26 27^ Stratified analysis was conducted by dementia status to examine the incidence of falls among these subpopulations. A ZINB model was applied due to overdispersion of the dependent variable and excessive zeros in our data.^28^ The strength of association was presented with adjusted incidence rate ratios (IRR) and incidence rate difference (IRD) with 95% CI. The IRR is relative, and the IRD is an absolute measure of effect.^29^ Data were analysed using the Stata software V.18.

## Results

### Participants

A total of 3064 unique residents were included in the study. The median age of the residents was 86 (IQR 80–90) years, and two-thirds (66.7%) were female. Over half of the residents had arthritis (55.7%) and dementia (51.3%). Table 1 describes the baseline characteristics of the residents.

**Table 1:** Baseline characteristics of residents from 23 residential aged care homes (2020–2021)

Characteristics	Total
N (%)	3064
Sex, n (%)	
Male	1019 (33.3)
Female	2045 (66.7)
Age, median (IQR)	86 (80–90)
Age category in years, n (%)	
65–74	380 (12.4)
75–84	979 (31.9)
85–94	1445 (47.2)
≥95	260 (8.48)
No. of medicines, n (%)	
1–4	487 (15.9)
5–8	1064 (34.7)
≥9	1513 (49.4)
Health conditions, n (%)	
Arthritis	1708 (55.7)
Dementia	1572 (51.3)
Cognitive impairment	1265 (41.3)
Fracture	1071 (34.9)
Anxiety	1023 (33.4)
Diabetes mellitus	802 (26.2)
Visual impairment	540 (17.6)
Delirium	372 (12.1)
Parkinson’s disease	173 (5.65)
Medicines (ATC level 1)	
Alimentary tract and metabolism (A)	2958 (96.5%)
Nervous system (N)	2867 (93.6)
Cardiovascular system (C)	2366 (77.2)
Anti-infectives for systemic use (J)	2253 (73.5)
Blood and blood-forming organs (B)	2070 (67.6)
Dermatologicals (D)	1861 (60.7)
Sensory organs (S)	1686 (55.0)
Musculoskeletal system (M)	1179 (38.5)
Respiratory system (R)	1065 (34.8)
Systematic hormonal preparations, excl. sex hormones and insulins (H)	869 (28.4)
Genitourinary system and sex hormones (G)	662 (21.6)
Antineoplastic and immunomodulating agents (L)	211 (6.88)
Antiparasitic products, insecticides, and repellents (P)	139 (4.53)
Various (V)	133 (4.34)

ATC, Anatomical Therapeutic Chemical.

### Exposure of residents to CNS-PIPMs

Online supplemental figure S1 displays the number of days residents were exposed to CNS-PIPMs and the proportion of days covered by CNS-PIPMs. Both metrics were calculated for overall CNS-PIPM usage as well as for different CNS-PIPM classes.

The overall median number of days residents were exposed to CNS-PIPMs was 91 days (IQR 6–320). The median number of days residents were exposed to CNS-PIPM classes ranged from 11 days (IQR 2–160) for benzodiazepines and Z drugs to 169 days (IQR 59–454) for antidepressants (online supplemental figure SA). The median proportion of days covered by at least one CNS-PIPM was 39.3% (IQR 2.6–86.6%) indicating that half of the residents received CNS-PIPM for 2 out of 5 days. The median proportion of days covered across the CNS-PIPM classes ranged from 5.7% (IQR 0.8–62.6) for benzodiazepines and Z drugs to 63.1% (IQR 19.3–91.3) for antidepressants (online supplemental figure SB).

### Incidence and characteristics of falls

There was a total of 17 946 falls reported for over 1 389 140 resident days. Of these, 34.3% (n=6173) were injurious falls, and 11.3% (n=2035) were falls requiring hospitalisation. At the resident level (n=3064), 64.9% (n=1989) experienced at least one fall, 47.9% (n=1469) injurious falls and 27.4% (n=841) falls requiring hospitalisation. The proportion of residents who experienced 2, 3, 4 and 5 falls were 9.9%, 7.8%, 6.1% and 5.00%, respectively, while 15.3% experienced >10 falls during the study period. Among residents who experienced a fall (n=1989), the median number of falls per resident was 2 (IQR 0–7), 1 (IQR 0–3) for injurious falls and 0 (IQR 0–1) for falls requiring hospitalisation.

### CNS-PIPMs and falls

Of the 3064 residents, 40% (n=1224) used at least one CNS-PIPM and 10% (n=302) used two or more CNS-PIPMs. The most frequently used CNS-PIPM categories were benzodiazepines and Z-drugs (27.4%), followed by first- and second-generation antipsychotics (17.2%) and antidepressants with strong anticholinergic activity (5.22%).

Of the 1224 residents using CNS-PIPMs, 70% (n=857) experienced at least one fall, 53.6% (n=656) injurious falls and 30.2% (n=370) falls requiring hospitalisation. The proportion of residents using CNS-PIPMs who experienced 2, 3, 4 and 5 falls were 9.5%, 8.2%, 6.1% and 5.00%, respectively, while 19.6% using CNS-PIPMs experienced >10 falls during the study period (online supplemental table S2). Table 2 shows a comparison of falls between residents who used a CNS-PIPM and those who did not. Residents using CNS-PIPMs experienced more falls as compared with non-users. Table 3 shows the falls comparison by dementia status between CNS-PIPM users and non-users. Residents using CNS-PIPMs experienced significantly more falls compared with non-users regardless of dementia status.

**Table 2:** Comparison of falls between CNS-PIPM users and non-users

	CNS-PIPM+N (%)	CNS-PIPM−N (%)	Total
All falls			
Yes	857 (70)	1132 (61.5)	1989 (64.9)
No	367 (30)	708 (38.5)	1075 (35.1)
Total	1224 (40)	1840 (60)	3064 (100)
Injurious falls			
Yes	656 (53.6)	813 (44.2)	1469 (47.9)
No	568 (46.4)	1027 (55.8)	1595 (52.1)
Total	1224 (40)	1840 (60)	3064 (100)
Falls requiring hospitalisation			
Yes	370 (30.2)	471 (25.6)	841 (27.4)
No	854 (69.8)	1369 (74.4)	2223 (72.6)
Total	1224 (40)	1840 (60)	3064 (100)

PIPM+ = PIPM users, PIPM-− = non-users.

CNS-PIPM, central nervous system psychotropic medicine.

**Table 3:** Falls comparison between CNS-PIPM users and non-users with and without dementia

	Dementia (n=1572)		Total	No dementia (n=1492)		Total	Overall
	CNS-PIPM+	CNS-PIPM−		CNS-PIPM+	CNS-PIPM−		
Any falls							
Yes	507 (75.6)	613 (68)	1120 (71.2)	350 (63.3)	519 (55.3)	869 (58.2)	1989 (64.9)
No	164 (24.4)	288 (32)	452 (28.8)	203 (36.7)	420 (44.7)	623 (41.8)	1075 (35.1)
Total	671 (42.7)	901 (57.3)	1572 (100)	553 (37.1)	939 (62.9)	1492 (100)	3064
Injurious falls							
Yes	395 (58.9)	459 (50.9)	854 (54.3)	261 (47.2)	354 (37.7)	615 (41.2)	1469 (47.9)
No	276 (41.1)	442 (49.1)	718 (45.7)	292 (52.8)	585 (62.3)	877 (58.8)	1595 (52.1)
Total	671 (42.7)	901 (57.3)	1572 (100)	553 (37.1)	939 (62.9)	1492 (100)	3064
Falls requiring hospitalisation							
Yes	217 (32.3)	255 (28.3)	472 (30)	153 (27.7)	216 (23.0)	369 (24.7)	841 (27.4)
No	454 (67.7)	646 (71.7)	1100 (70)	400 (72.3)	723 (77.0)	1123 (75.3)	2223 (72.6)
Total	671 (42.7)	901 (57.3)	1572 (100)	553 (37.1)	939 (62.9)	1492 (100)	3064

CNS-PIPM+ = PIPM users, CNS-PIPM− = non-users

CNS-PIPM, central nervous system psychotropic medicine.

### Fall incident rates between CNS-PIPM users and non-users

**Figure 1:**
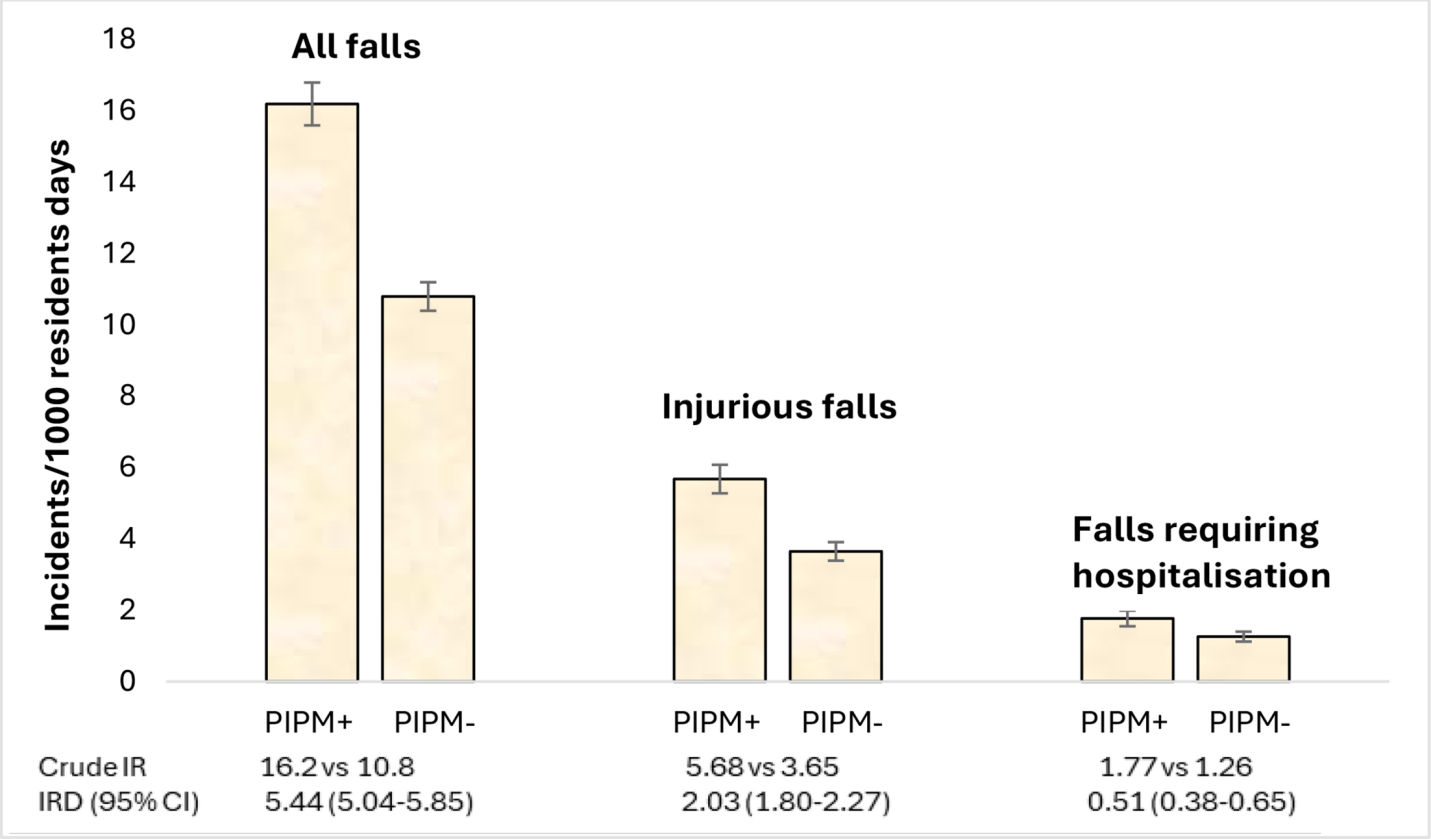
Comparisons of fall incidents by CNS-PIPM+ and CNS-PIPM−. The error bars represent 95% CI. CNS-PIPM+ = PIPM users, CNS-PIPM− = non-users. CNS-PIPM, central nervous system psychotropic medicine; IRD, incidence rate difference.

The overall falls IRs were 12.9 falls per 1000 resident days (95% CI 12.7 to 13.1), 4.44 falls per 1000 resident days (95% CI 4.33 to 4.55) and 1.46 falls per 1000 resident days (95% CI 1.40 to 1.53) for all falls, injurious falls and falls requiring hospitalisation, respectively. The falls IRs of CNS-PIPM users were 16.2 falls per 1000 resident days (95% CI 15.9 to 16.5), 5.68 falls per 1000 resident days (95% CI 5.48 to 5.88) and 1.77 falls per 1000 resident days (95% CI 1.66 to 1.88) for all falls, injurious falls and falls requiring hospitalisation respectively. The falls IRs of non-CNS-PIPM users were 10.8 falls per 1000 resident days (95% CI 10.6 to 11.0), 3.65 falls per 1000 resident days (95% CI 3.52 to 3.78) and 1.26 falls per 1000 resident days (95% CI 1.19 to 1.33) for all falls, injurious falls and falls requiring hospitalisation, respectively. Figure 1 compares the IRs of CNS-PIPM users and non-users. The IRs of falls were greater in CNS-PIPM users compared with non-users. The IR of all falls for CNS-PIPM users was 1.29 times the IR for non-users (95% CI 1.16 to 1.44) with an IRD of 5.44 falls per 1000 resident days (95% CI 5.04 to 5.85). For injurious falls, the IR for CNS-PIPM users was 1.35 times the IR for non-users (95% CI 1.21 to 1.50) with an IRD of 2.03 falls per 1000 resident days (95% CI 1.80 to 2.27). The IR of falls requiring hospitalisation for CNS-PIPM users was 1.21 times the IR for non-users (95% CI 1.06 to 1.38) with an IRD of 0.51 falls per 1000 resident days (95% CI 0.38 to 0.65). Table 4 presents the results of a ZINB regression analysis comparing the incidence of falls for CNS-PIPM users and non-users with and without dementia. CNS-PIPM users had a significantly greater rate of falls compared with non-users regardless of dementia status for all outcomes. Online supplemental table S3 shows factors associated with the number of falls experienced by older adults living in residential aged care.

**Table 4:** Incidence of falls of CNS-PIPM users and non-users with and without dementia

	Overall	Dementia status	
		Dementia (n=1572)	No dementia (n=1492)
	Adjusted IRR(95% CI)	Adjusted IRR(95% CI)	Adjusted IRR(95% CI)
All falls			
CNS-PIPM+ vs CNS-PIPM− (ref=CNS-PIPM−)	1.29 (1.16 to 1.44)*	1.33 (1.16 to 1.54)*	1.24 (1.05 to 1.45)*
Injurious falls			
CNS-PIPM+ vs CNS-PIPM− (ref=CNS-PIPM−)	1.35 (1.21 to 1.50)*	1.36 (1.18 to 1.57)*	1.30 (1.10 to 1.55)*
Falls requiring hospitalisation			
CNS-PIPM+ vs CNS-PIPM− (ref=CNS-PIPM−)	1.21 (1.06 to 1.38)*	1.20 (1.01 to 1.43)*	1.23 (1.01 to 1.51)*

All analyses were adjusted for age, sex, number of medicines, health conditions and ATC level 1 medicines presented in table 1 using zero-inflated negative binomial regression modelling. CNS-PIPM+ = PIPM users, CNS-PIPM− = non-users.

* p<0.05

CNS-PIPM, central nervous system psychotropic medicine; IRR, incidence rate ratio.

## Discussion

### Statement of principal findings

This longitudinal, multi-facility study among 3064 older adults provides new evidence of the association between falls and CNS-PIPMs in RACFs in Australia. Our study showed that falls were very common among older adults using CNS-PIPMs, with 70% of residents falling at least once. For every 1000 resident days, there were 16 falls of any type, 6 injurious falls and 2 falls resulting in hospitalisation among CNS-PIPM users. CNS-PIPM users had a five times higher risk of falling compared with non-users.

### Interpretation within the context of the wider literature

Our study used the updated Beers criteria 2023 and specifically focuses on the use of potentially inappropriate medicines related to CNS medications. The data used in our study were from the time period 2020–2021, and Beer criteria were updated in 2023. Comparisons with the previous studies that may have used the Beers criteria 2019 remain valid, but the changes regarding CNS medicines in the updated version 2023 do not substantially impact the overall proportion of potentially inappropriate medicines in our population, regardless of whether the 2019 or 2023 criteria were applied. The removal of CNS medicines from the 2023 version (eg, protriptyline, trimipramine, amobarbital) does not affect the extent of potentially inappropriate medicines use as these medicines were also discontinued in the Australian market^30^ and were not available for use during the period of study (2020–2021). Furthermore, the addition of potentially inappropriate anti-Parkinson’s drugs in the 2023 version showed minimal contribution in our study.

We found more falls were experienced by residents who were CNS-PIPM users compared with non-users. Prior research has documented an increase in fall incidents with the use of psychotropic medicine, that is, a study of 2368 older adults from 9 RACFs in the Netherlands found a threefold increase in falls among psychotropic medicine users compared to non-users (OR 2.88; 95% CI 1.52 to 5.44) over 2 years.^31^ Another study among 4502 residents from 41 nursing homes in Spain found that residents on psychotropic medicines experienced falls, with 8.8% of them having at least one fall during the 1 month study period.^32^ However, both these studies were cross-sectional, used medication prescription data with the assumption that the medication was administered, and did not explore differences between types of falls. Unlike previous studies, our study used a retrospective longitudinal cohort design with an administrative dataset and evaluated three fall groups (any falls, injurious falls and falls requiring hospitalisation). Psychotropic medicines act directly on CNS, and their psychomotor effects such as sedation, orthostatic hypotension, dizziness, confusion and sleep disturbance may increase the risk of falls among older adults.^33^

Previous studies have identified the risk of adverse outcomes (morbidity, mortality, functional decline) among older adults with the use of potentially inappropriate medicines^19 34^ and specifically the association of fall risks with psychotropic medicines.^35^ A recent systematic review conducted in France reported increased risk of falls among PIPM users compared with non-users (OR 1.23; 95% CI 1.15 to 1.32), among older people in the community and nursing homes.^36^ We showed a similar result but with a wider range of fall outcomes.

Facilities with a higher proportion of CNS-PIPM users will likely experience a higher rate of falls. Behavioural disturbances in dementia often stem from a range of triggers, including environmental changes, unmet needs (such as hunger or discomfort), overstimulation, stress, inconsistent routines, poor communication and unfamiliar faces.^37^ These factors can contribute to confusion, agitation or aggression, potentially leading to the use of psychotropic medications. While such medications may sometimes be necessary, current best practice guidelines^38 39^ emphasise that non-pharmacological interventions should be the first-line approach for managing behavioural and psychological symptoms of dementia (BPSD). Psychotropic medications, including antipsychotics, benzodiazepines and antidepressants, should be considered only when behavioural symptoms pose significant distress or harm and when non-pharmacological strategies such as cognitive behavioural therapy, psychosocial interventions, physical activity and other sensory simulation and music therapies^40^ have been ineffective. In addition, high benzodiazepine use may increase the risk of falls, fractures, anxiety disorders, pneumonia and dementia.^41^,^43^ Inappropriate antipsychotic use can lead to pneumonia, venous thromboembolism and cerebrovascular events,^44 45^ while inappropriate antidepressants may heighten the risk of cognitive deficits.^46^ Given these risks, careful consideration and regular review of psychotropic prescriptions are essential to ensure appropriate and safe use in older adults with dementia.^47^ Regular use of PIPM should be avoided, and deprescribing of psychotropic medicines should be encouraged where possible. Individualised treatment plans, for example, using therapeutic decision trees modified by an individual’s and environmental risk profile, have been recommended.^48^

### Policy implications

The results of our study have important policy implications for improved fall prevention and management for PIPM users in RACF settings. The high fall rates among CNS-PIPM users compared with non-users emphasise that PIPMs should be given attention when developing falls prevention and intervention programmes. Psychotropic medicine use has increased to 60% in the past 30 years for all Australians.^49^ Appropriate use of psychotropic medicines is significantly less harmful compared with inappropriate use.^50^ Thus, a high frequency of falls and related injuries and hospitalisation with CNS-PIPM use confirms the need for close monitoring and reviewing psychotropic medicine regularly, ensuring an assessment of the relative risks and benefits to residents. According to recent guidelines on fall prevention and management among older adults, medicines should be reviewed at least once a year for all older adults and every 6 months among frail residents.^51^

### Strengths and limitations

The study’s strength lies in its multicentre design, involving a substantial cohort of 3064 residents from 23 different RACFs and the use of a comprehensive measure of falls (three types of falls) among CNS-PIPM users and non-users. These factors enhance the study’s statistical power and generalisability of findings, allowing for a more robust and representative examination of the impact of CNS-PIPM on falls. Moreover, this study used data about administered medicines rather than prescribed medicines to capture actual use of medicines.

A limitation of the study is that the results focus on RACFs from one provider and therefore may not be representative of all RACFs in Australia. Furthermore, we did not have any information about the reasons for CNS-PIPM use by residents. We evaluated the medicines flagged as potentially inappropriate in the Beers criteria 2023, but we did not have detailed clinical information about each resident and therefore could not confirm whether they were indeed misused or actually inappropriate. Moreover, the conditions that lead to prescribing psychotropic medications such as agitation, restlessness, confusion, aggression or sleep disturbances may themselves increase the risk of falls, but we didn’t consider these variables due to lack of data on these variables. In addition to this, we could not find the dose–response relationship to know if the risk of falls increased with the number of administered potentially inappropriate psychotropic medicines because of limited clinical information in our dataset.

## Conclusion

The present study revealed that falls are frequent among CNS-PIPM users resulting in injury and hospitalisation, with 70% of CNS-PIPM users falling at least once, and one in three requiring admissions in hospital. This supports recommendations to regularly review psychotropic medicine use and ensure its use is considered when developing falls prevention programmes.

## Supplementary material

10.1136/bmjopen-2024-096187 online supplemental file 1

## Data Availability

Data are available upon reasonable request.

## References

[1] Mattig T (2020). Falls in the elderly: a major public health challenge with some encouraging developments. A mini review. JGG.

[2] Mekkodathil A, El-Menyar A, Kanbar A (2020). Epidemiological and clinical characteristics of fall-related injuries: a retrospective study. BMC Public Health.

[3] Berková M, Berka Z (2018). Falls: a significant cause of morbidity and mortality in elderly people. Vnitr Lek.

[4] World Health Organization (2021). Falls. https://www.who.int/news-room/fact-sheets/detail/falls.

[5] Mays AM (2024). Geriatric medicine.

[6] Cameron EJ, Bowles SK, Marshall EG (2018). Falls and long-term care: a report from the care by design observational cohort study. BMC Fam Pract.

[7] Australian Institute of Health Welfare (2023.). Injury in Australia: falls. https://www.aihw.gov.au/reports/injury/falls.

[8] Pointer S, Harrison J, Avefua S (2019). Trends in hospitalised injury due to falls in older people 2007–08 to 2016–17.

[9] Wabe N, Seaman KL, Nguyen AD (2022). Epidemiology of falls in 25 Australian residential aged care facilities: a retrospective longitudinal cohort study using routinely collected data. Int J Qual Health Care.

[10] Cameron ID, Dyer SM, Panagoda CE (2018). Interventions for preventing falls in older people in care facilities and hospitals. Cochrane Database Syst Rev.

[11] Colón-Emeric CS, McDermott CL, Lee DS (2024). Risk Assessment and Prevention of Falls in Older Community-Dwelling Adults: A Review. JAMA.

[12] Pasquetti P, Apicella L, Mangone G (2014). Pathogenesis and treatment of falls in elderly. Clin Cases Miner Bone Metab.

[13] Lind KE, Raban MZ, Brett L (2020). Measuring the prevalence of 60 health conditions in older Australians in residential aged care with electronic health records: a retrospective dynamic cohort study. Popul Health Metr.

[14] Stephenson CP, Karanges E, McGregor IS (2013). Trends in the utilisation of psychotropic medications in Australia from 2000 to 2011. Aust N Z J Psychiatry.

[15] Osman A, Kamkar N, Speechley M (2022). Fall risk-increasing drugs and gait performance in community-dwelling older adults: A systematic review. Ageing Res Rev.

[16] Ćurković M, Dodig-Ćurković K, Erić AP (2016). Psychotropic medications in older adults: a review. Psychiatr Danub.

[17] Azab M, Novella A, Ianes A (2024). Potentially Inappropriate Psychotropic Drugs in Nursing Homes: An Italian Observational Study. Drugs Aging.

[18] Lind KE, Raban MZ, Georgiou A (2019). Duration of Antipsychotic Medication Use by Aged Care Facility Residents With Dementia. Alzheimer Dis Assoc Disord.

[19] Xing XX, Zhu C, Liang HY (2019). Associations Between Potentially Inappropriate Medications and Adverse Health Outcomes in the Elderly: A Systematic Review and Meta-analysis. Ann Pharmacother.

[20] Ryan-Atwood TE, Hutchinson-Kern M, Ilomäki J (2017). Medication Use and Fall-Related Hospital Admissions from Long-Term Care Facilities: A Hospital-Based Case-Control Study. Drugs Aging.

[21] Hien LTT, Cumming RG, Cameron ID (2005). Atypical antipsychotic medications and risk of falls in residents of aged care facilities. J Am Geriatr Soc.

[22] Wang KN, Bell JS, Gilmartin-Thomas JFM (2021). Use of Falls Risk Increasing Drugs in Residents at High and Low Falls Risk in Aged Care Services. J Appl Gerontol.

[23] Wabe N, Huang G, Silva SM (2024). A Longitudinal Study of the Use and Effects of Fall-Risk-Increasing Drugs in Residential Aged Care. J Am Med Dir Assoc.

[24] WHOCC (2018.). Purpose of the ATC/DDD system. https://www.whocc.no/atc_ddd_methodology/purpose_of_the_atc_ddd_system.

[25] By the 2023 American Geriatrics Society Beers Criteria Update Expert Panel (2023). American Geriatrics Society 2023 updated AGS Beers Criteria for potentially inappropriate medication use in older adults. J Am Geriatr Soc.

[26] Shao L, Shi Y, Xie X-Y (2023). Incidence and Risk Factors of Falls Among Older People in Nursing Homes: Systematic Review and Meta-Analysis. J Am Med Dir Assoc.

[27] Enderlin C, Rooker J, Ball S (2015). Summary of factors contributing to falls in older adults and nursing implications. Geriatr Nurs.

[28] Greene WH (1994). Accounting for excess zeros and sample selection in Poisson and negative binomial regression models.

[29] Tripepi G, Jager KJ, Dekker FW (2007). Measures of effect: relative risks, odds ratios, risk difference, and 'number needed to treat'. Kidney Int.

[30] (2024.). Australian medicines handbook. https://amhonline-amh-net-au.simsrad.net.ocs.mq.edu.au.

[31] Cox CA, van Jaarsveld HJ, Houterman S (2016). Psychotropic Drug Prescription and the Risk of Falls in Nursing Home Residents. J Am Med Dir Assoc.

[32] Olazarán J, Valle D, Serra JA (2013). Psychotropic medications and falls in nursing homes: a cross-sectional study. J Am Med Dir Assoc.

[33] Glab KL, Wooding FGG, Tuiskula KA (2014). Medication-related falls in the elderly: mechanisms and prevention strategies. Consult Pharm.

[34] Hyttinen V, Jyrkkä J, Valtonen H (2016). A Systematic Review of the Impact of Potentially Inappropriate Medication on Health Care Utilization and Costs Among Older Adults. Med Care.

[35] Johnell K, Jonasdottir Bergman G, Fastbom J (2017). Psychotropic drugs and the risk of fall injuries, hospitalisations and mortality among older adults. Int J Geriatr Psychiatry.

[36] Corvaisier M, Brangier A, Annweiler C (2024). Preventable or potentially inappropriate psychotropics and adverse health outcomes in older adults: systematic review and meta-analysis. J Nutr Health Aging.

[37] Ceppi L (2024). Caring for older adults: bio-psycho-social assessment and well-being in nursing homes.

[38] Gray KL, Moniz-Cook E, Reichelt K (2022). Professional perspectives on applying the NICE and British Psychological Society Guidelines for the management of Behaviours that Challenge in dementia care: an e-survey. Br J Clin Psychol.

[39] Bokhari SA, Qassem T, Al-Ayyat D (2025). The 2024 clinical practice guidelines on the pharmacological management of Behavioural and Psychological Symptoms of Dementia (BPSD) in the Arab world. Middle East Curr Psychiatry.

[40] Dementia Centre for Research Collaboration (DCRC) (2022). Behaviour management: a guide to good practice, managing behavioural and psychological symptoms of dementia.

[41] Sterke CS, van Beeck EF, van der Velde N (2012). New insights: dose-response relationship between psychotropic drugs and falls: a study in nursing home residents with dementia. J Clin Pharmacol.

[42] Taipale H, Tolppanen A-M, Koponen M (2017). Risk of pneumonia associated with incident benzodiazepine use among community-dwelling adults with Alzheimer disease. CMAJ.

[43] Billioti de Gage S, Bégaud B, Bazin F (2012). Benzodiazepine use and risk of dementia: prospective population based study. BMJ.

[44] Harrison F, Cations M, Jessop T (2020). Prolonged use of antipsychotic medications in long-term aged care in Australia: a snapshot from the HALT project. Int Psychogeriatr.

[45] Rogowska M, Thornton M, Creese B (2023). Implications of Adverse Outcomes Associated with Antipsychotics in Older Patients with Dementia: A 2011-2022 Update. Drugs Aging.

[46] Heser K, Luck T, Röhr S (2018). Potentially inappropriate medication: Association between the use of antidepressant drugs and the subsequent risk for dementia. J Affect Disord.

[47] Batool N, Raban MZ, Seaman KL (2024). Use of potentially inappropriate psychotropic medicines among older adults in 23 residential aged care facilities in Australia: a retrospective cohort study. BMC Geriatr.

[48] Tible OP, Riese F, Savaskan E (2017). Best practice in the management of behavioural and psychological symptoms of dementia. Ther Adv Neurol Disord.

[49] Australian Commission on Safety and Quality in Health Care (2024). Mindful use of psychotropic medicines: new standard a lifeline for people with cognitive disability or impairment. https://www.safetyandquality.gov.au/newsroom/latest-news/mindful-use-psychotropic-medicines-new-standard-lifeline-people-cognitive-disability-or-impairment.

[50] Hiance-Delahaye A, de Schongor FM, Lechowski L (2018). Potentially inappropriate prescription of antidepressants in old people: characteristics, associated factors, and impact on mortality. Int Psychogeriatr.

[51] Montero-Odasso M, van der Velde N, Martin FC (2022). World guidelines for falls prevention and management for older adults: a global initiative. Age Ageing.

