# Supplementary material for: Impact of potentially inappropriate psychotropic medicines on falls among older adults in 23 residential aged care facilities in Australia: a retrospective longitudinal cohort study
Source: BMJ Open. 2025 Apr 9;15(4):e096187. doi: 10.1136/bmjopen-2024-096187 (PMC12004465; doi:10.1136/bmjopen-2024-096187)
Supplement: online supplemental file 1 [file bmjopen-15-4-s001.docx]

**Supplementary file**

**Table S1 Beers criteria (2023) for potentially inappropriate psychotropic medicines (PIPMs)**

| **Drug class** | **Specific medications considered** | **Recommendation** | **ATC codes** |
| --- | --- | --- | --- |
| Antidepressants with strong anticholinergic activity | Amitriptyline Amoxapine Clomipramine Desipramine Doxepin Imipramine Nortriptyline Paroxetine | Avoid | N06A |
| Antiparkinsonian agents with strong anticholinergic activity | Benztropine Trihexyphenidyl | Avoid | N04A |
| Antipsychotics first- (typical) and second- (atypical) generation | Amisulpride  Aripiprazole  Brexpiprazole  Cariprazine Chlorpromazine Clozapine  Flupenthixol  Fluphenazine Haloperidol  Levomepromazine  Lurasidone  Olanzapine  Paliperidone  Pericyazine  Perphenazine  Pimavanserin  Prochlorperazine Quetiapine Risperidone  Ziprasidone  Zuclopenthixol | Avoid, except in FDA-approved indications such as schizophrenia, bipolar disorder. | N05A |
| Barbiturates | Butalbital Phenobarbital Primidone | Avoid | N03A |
| Benzodiazepines | Alprazolam Chlordiazepoxide Clobazam Clonazepam Clorazepate Diazepam Estazolam Lorazepam Midazolam Oxazepam Temazepam Triazolam | Avoid | N05B |
| Nonbenzodiazepine benzodiazepine receptor agonist hypnotics (“Z-drugs”) | Eszopiclone Zaleplon Zolpidem | Avoid | N05C |

**Table S2 Number of falls experienced by residents using CNS-PIPMs during the study period**

| Number  of falls | Any falls | | Injurious falls | | Falls requiring hospitalisation | |
| --- | --- | --- | --- | --- | --- | --- |
|  | n | % | n | % | n | % |
| 0 | 256 | 20.9 | 439 | 35.9 | 739 | 60.4 |
| 1 | 161 | 13.1 | 247 | 20.2 | 259 | 21.2 |
| 2 | 116 | 9.48 | 151 | 12.3 | 112 | 9.15 |
| 3 | 100 | 8.17 | 97 | 7.92 | 56 | 4.58 |
| 4 | 74 | 6.05 | 80 | 6.54 | 24 | 1.96 |
| 5 | 61 | 4.98 | 50 | 4.08 | 11 | 0.90 |
| 6 | 59 | 4.82 | 38 | 3.10 | 12 | 0.98 |
| 7 | 47 | 3.84 | 24 | 1.96 | 5 | 0.41 |
| 8 | 46 | 3.76 | 19 | 1.55 | 1 | 0.08 |
| 9 | 41 | 3.35 | 14 | 1.14 | 3 | 0.25 |
| 10 | 23 | 1.88 | 12 | 0.98 | 0 | 0.00 |
| >10 | 240 | 19.61 | 53 | 4.33 | 2 | 0.16 |

**Table S3** **Factors associated with the number of falls experienced by older adults living in residential aged care**

|  | **Any falls** | **Injurious falls** | **Falls requiring hospitalisation** |
| --- | --- | --- | --- |
|  | **Adjusted**  IRR (95% CI) | **Adjusted**  IRR (95% CI) | **Adjusted**  IRR (95% CI) |
| **CNS-PIPMs** [Ref=CNS-PIPM-] |  |  |  |
| CNS-PIPMs+ vs CNS-PIPMs- | 1.29 (1.16-1.44)* | 1.35 (1.21-1.50)* | 1.21 (1.06-1.38)* |
| **Sex** [Ref=Male] |  |  |  |
| Female vs Male | 0.76 (0.68-0.85)* | 0.71 (0.63-0.80)* | 0.87 (0.76-1.01) |
| **Age** [Ref=65-74 years] |  |  |  |
| 75-84 | 0.99 (0.83-1.17) | 1.11 (0.93-1.33) | 1.19 (0.95-1.49) |
| 85-94 | 1.05 (0.89-1.24) | 1.43 (1.20-1.70)* | 1.44 (1.16-1.79)* |
| ≥95 | 1.02 (0.81-1.29) | 1.55 (1.22-1.97)* | 1.34 (0.99-1.82) |
| **No. of medicines** [Ref=1-4] |  |  |  |
| 5-8 | 1.31 (1.12-1.53)* | 1.29 (1.09-1.52)* | 1.21 (0.98-1.48) |
| >=9 | 1.45 (1.24-1.69)* | 1.51 (1.28-1.77)* | 1.44 (1.18-1.75)* |
| **Health status** |  |  |  |
| Arthritis | 1.16 (1.05-1.29)* | 1.14 (1.03-1.28)* | 1.17 (1.03-1.33)* |
| Dementia | 2.21 (1.99-2.46)* | 1.94 (1.74-2.16)* | 1.59 (1.39-1.82)* |
| Cognitive impairment | 1.22 (1.10-1.36)* | 1.20 (1.07-1.33)* | 1.20 (1.05-1.37)* |
| Fracture | 1.49 (1.34-1.66)* | 1.63 (1.46-1.82)* | 1.71 (1.50-1.94)* |
| Anxiety | 1.15 (1.03-1.28)* | 1.19 (1.06-1.33)* | 1.15 (1.00-1.31) |
| Diabetes mellitus | 0.90 (0.80-1.01) | 0.86 (0.76-0.97)* | 1.03 (0.89-1.19) |
| Visual impairment | 1.16 (1.02-1.33)* | 1.11 (0.97-1.27) | 1.09 (0.92-1.28) |
| Delirium | 1.10 (0.95-1.29) | 1.04 (0.89-1.22) | 1.06 (0.88-1.28) |
| Parkinson’s disease | 1.87 (1.50-2.33)* | 1.67 (1.34-2.09)* | 1.29 (0.99-1.69) |
| **Medicines** |  |  |  |
| Alimentary tract and metabolism (A) | 1.11 (0.90-1.36) | 1.13 (0.91-1.41) | 0.96 (0.74-1.25) |
| Nervous system (N) | 1.05 (0.85-1.30) | 1.12 (0.90-1.39) | 1.08 (0.83-1.41) |
| Cardiovascular system (C) | 0.85 (0.68-1.07) | 0.89 (0.70-1.12) | 0.84 (0.64-1.12) |
| Antiinfectives for systemic use (J) | 1.26 (0.79-2.01) | 1.39 (0.86-2.24) | 1.17 (0.65 -2.08) |
| Blood and blood forming organs (B) | 1.03 (0.79 -1.36) | 1.04 (0.78-1.38) | 1.00 (0.72 -1.41) |
| Dermatologicals (D) | 1.02 (0.75-1.38) | 1.21 (0.88-1.66) | 1.09 (0.75-1.58) |
| Sensory organs (S) | 1.01 (0.76 -1.33) | 0.92 (0.69-1.24) | 0.83 (0.58-1.19) |
| Musculo-skeletal system (M) | 1.41 (0.97-2.05) | 1.27 (0.86-1.88) | 0.96 (0.60-1.55) |
| Respiratory system (R) | 1.05 (0.73-1.50) | 1.27 (0.87-1.84) | 1.25 (0.81-1.94) |
| Systematic hormonal preparations, excl. sex hormones and insulins (H) | 1.28 (0.89-1.84) | 1.44 (1.00-2.09) | 0.97 (0.61-1.53) |
| Genito urinary system and sex hormones (G) | 0.74 (0.47-1.18) | 0.86 (0.53-1.39) | 0.72 (0.39-1.33) |
| Antineoplastic and immunomodulating agents (L) | 0.58 (0.23-1.46) | 0.71 (0.26-1.95) | 0.89 (0.27-2.97) |
| Antiparasitic products, insecticides and repellents (P) | 0.20 (0.01-5.22) | 0.00 (0.00) | 0.00 (0.00) |
| Various (V) | 0.83 (0.24-2.88) | 1.32 (0.38-4.58) | 1.58 (0.41-6.04) |

**SA**


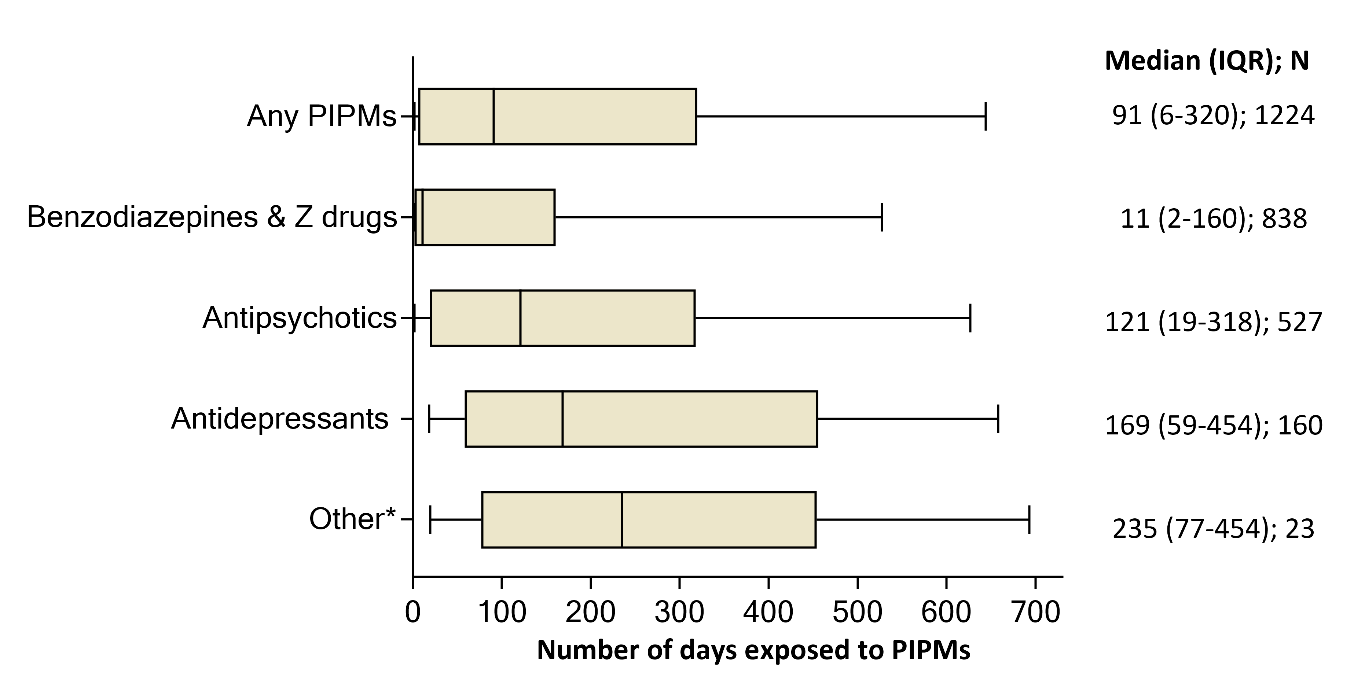


**SB**


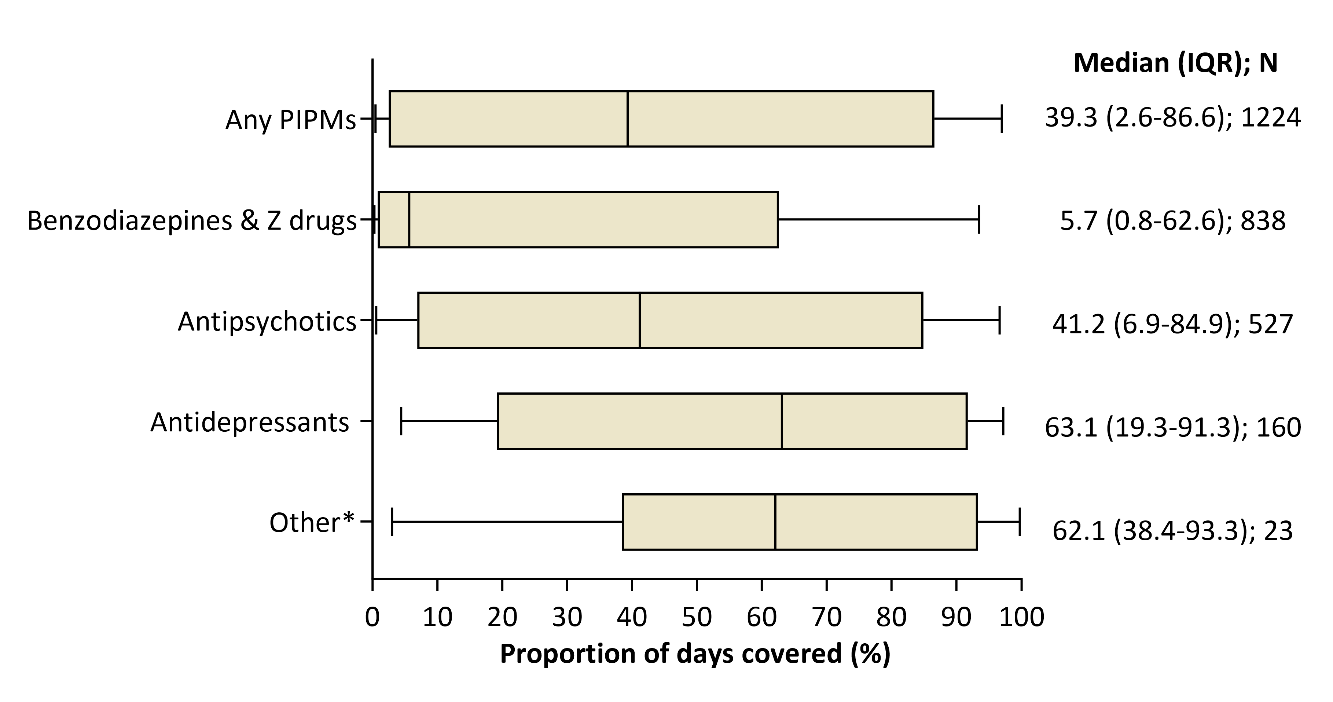


**Figure S1: The number of days residents were exposed to CNS-PIPMs and proportion of days covered by CNS-PIPMs** *Includes antiparkinsonian agents (n=17) and barbiturates (n=6). Boxes in the figure represent the IQR with the median value within the boxes and the capped bars represent the 10th and 90th percentiles. PIPM= CNS-PIPMs. Number of days residents were exposed to CNS-PIPMs represent the total number of days residents received a specific CNS-PIPM during their stay, while proportion of days covered by CNS-PIPMs is the ratio of number of days residents were exposed to CNS-PIPMs to the resident's length of stay.
