# Supplementary material for: Impact of potentially inappropriate psychotropic medicines on falls among older adults in 23 residential aged care facilities in Australia: a retrospective longitudinal cohort study
Source: BMJ Open. 2025 Apr 9;15(4):e096187. doi: 10.1136/bmjopen-2024-096187 (PMC12004465; doi:10.1136/bmjopen-2024-096187)
Supplement: Supplementary file 2 [file bmjopen-2024-096187.reviewer_comments.pdf]

PEER REVIEW HISTORY

BMJ Open publishes all reviews undertaken for accepted manuscripts. Reviewers are asked to complete a checklist review form and are provided with free text boxes to elaborate on their assessment. These free text comments are reproduced below.

ARTICLE DETAILS

Title (Provisional)

Impact of potentially inappropriate psychotropic medicines on falls among older adults in 23 residential aged care facilities in Australia: a retrospective longitudinal cohort study

Authors

Batool, Narjis; Raban, Magdalena Z; Seaman, Karla; Westbrook, Johanna; Wabe, Nasir

---

VERSION 1 - REVIEW

**Reviewer**	**1**
**Name**	**Tan, Maw Pin**
**Affiliation**	**Faculty of Medicine, Department of Medicine**
**Date**	**21-Dec-2024**
**COI**	**None**

The authors conducted a longitudinal analysis of older persons in Residential Aged Care Facilities with and without exposure of inappropriately prescribed psychotropics identified using the Beer's criteria for CNS drugs. They found that those with inappropriately prescribed psychotropics were more likely to fall. This is an important finding considering that inappropriate psychotropic use in residential aged care facilities remains a major issue, and demonstration of significant negative effects will help increase the evidence-based to back up future efforts to curb this practice.

There are, however, a few concerns:

1. The comparison between those with and without inappropriate psychotropics currently does not seem to take into account the possibility that indications for psychotropic prescribing themselves may also lead to increase the risk of falls. Hence the authors to do seem to have addressed this by accounting for such potential confounding effects.

2. The authors utilized the Beer's criteria, but only evaluated CNS drugs. Could they justify why the had limited the evaluation for potentially inappropriate prescribing just for CNS drugs since Beer's also addresses other drugs classes, and these may also lead to increased risk of falls.

3. While the authors have suggested that psychotropics prescription in older adults may be

inappropriate, it is unclear why how they then concluded that psychotropics should be prescribed second line for the management of behavioural disturbance. The authors should instead consider providing some explanation of the current understanding on triggers for behavioural disturbance for dementia. There should also be some description of the cardiovascular risk association with prescribing antipsychotics.

Specific comments have be added within the pdf document attached.

The reviewer provided a marked copy with additional comments. Please contact the publisher for full details.

**Reviewer**	**2**
**Name**	**Agustí , Antonia**
**Affiliation**	**Hospital Universitari Vall d'Hebron, Clinical Pharmacology**
**Date**	**31-Jan-2025**
**COI**	**None**

Manuscript ID bmjopen-2024-096187, entitled "Impact of potentially inappropriate psychotropic medicines on falls among older adults in 23 residential aged care facilities in Australia: a retrospective longitudinal cohort study."

The authors analysed the possible relationship between treatment with potential inappropriate psychotropic medicines and increase of risk of falls in old people living in residential age care facilities in Australia. Although this is not a new issue, the article adds as a novelty information data on the different kind subtypes of falls and, in my opinion, it is, in general, methodologically well designed.

I only have on major concern and it is about the duration of treatment, did the authors take into consideration for how long these potential inappropriate psychotropic medicines were administered? Did the authors consider any minimum duration of treatment for each potential inappropriate psychotropic drug?

Did the authors analyse if the risk of falls increased with the number of administered potentially inappropriate psychotropic medicines?

VERSION 1 - AUTHOR RESPONSE

Reviewer #1:

Dr. Maw Pin Tan, Faculty of Medicine
Comments to the Author:
The authors conducted a longitudinal analysis of older persons in Residential Aged Care Facilities with and without exposure of inappropriately prescribed psychotropics identified using the Beer's criteria for CNS drugs. They found that those with inappropriately prescribed psychotropics were more likely to fall. This is an important finding considering that inappropriate psychotropic use in residential aged care facilities remains a major issue, and demonstration of significant negative

effects will help increase the evidence-based to back up future efforts to curb this practice.
There are, however, a few concerns:

Comment 1

The comparison between those with and without inappropriate psychotropics currently does not seem to take into account the possibility that indications for psychotropic prescribing themselves may also lead to increase the risk of falls. Hence the authors to do seem to have addressed this by accounting for such potential confounding effects.

Response:

Thank you for this comment. In our dataset limited clinical details were available and we didn't consider agitation, restlessness, confusion, aggression, or sleep disturbances due to lack of data on these variables. In the light of reviewer comment we have added the following in the limitation section (part of discussion) of the revised manuscript.

"The conditions that lead to prescribing psychotropic medications such as agitation, restlessness, confusion, aggression, or sleep disturbances may themselves increase the risk of falls, but we didn't consider these variables due to lack of data on these variables". (page 13-14 lines 326-329)

Comment 2

The authors utilized the Beer's criteria but only evaluated CNS drugs. Could they justify why the had limited the evaluation for potentially inappropriate prescribing just for CNS drugs since Beer's also addresses other drugs classes, and these may also lead to increased risk of falls.

Response:

Thank you for this comment and for highlighting this important consideration. We acknowledge that the Beers Criteria encompass a wide range of potentially inappropriate medications beyond CNS drugs. We have used only the CNS drug category from Beers criteria because of the following reason:

Other drug classes in the first category of Beers criteria require more detailed clinical information, which was not suitable for our dataset where limited clinical details were available. CNS drug classes were included because they require less clinical information. We have added the following text in the methods section of the revised manuscript.

"Our decision to focus specifically on CNS drugs was guided by the availability of clinical information within our dataset. Many drug classes included in the first category of the Beers Criteria require additional clinical details such as laboratory values, diagnostic history, and comorbid conditions that were not comprehensively captured in our dataset. In contrast, CNS drugs could be assessed with the available data without the need for extensive additional clinical context. Given the strong evidence linking CNS medications to fall risk, we prioritised evaluating this group to ensure robust and meaningful findings within the constraints of our dataset". (page 4 lines 109-116)

Comment 3

While the authors have suggested that psychotropics prescription in older adults may be inappropriate, it is unclear why how they then concluded that psychotropics should be prescribed second line for the management of behavioural disturbance. The authors should instead consider providing some explanation of the current understanding on triggers for behavioural disturbance for dementia. There should also be some description of the cardiovascular risk association with prescribing antipsychotics.

Response:

Thank you for your insightful comment. We appreciate the opportunity to clarify our rationale and provide additional context regarding the prescription of psychotropic medications for behavioural disturbances in dementia. We also acknowledge the need to discuss the risks associated with psychotropic medication use in older adults. In the light of reviewer comment we have added the following in the discussion section of the revised manuscript.

"Behavioural disturbances in dementia often stem from a range of triggers, including environmental changes, unmet needs (such as hunger or discomfort), overstimulation, stress, inconsistent routines, poor communication, and unfamiliar faces [37]. These factors can contribute to confusion, agitation, or aggression, potentially leading to the use of psychotropic medications. While such medications may sometimes be necessary, current best practice guidelines [38, 39] emphasise that non-pharmacological interventions should be the first-line approach for managing behaviours and psychological symptoms of dementia (BPSD). Psychotropic medications, including antipsychotics, benzodiazepines, and antidepressants, should be considered only when behavioural symptoms pose significant distress or harm and when non-pharmacological strategies have been ineffective. In addition, high benzodiazepine use may increase the risk of falls, fractures, anxiety disorders, pneumonia, and dementia [41-43]. Inappropriate antipsychotic use can lead to pneumonia, venous thromboembolism, and cerebrovascular events [44, 45], while inappropriate antidepressants may heighten the risk of cognitive deficits [46]. Given these risks, careful consideration and regular review of psychotropic prescriptions are essential to ensure appropriate and safe use in older adults with dementia". (page 12 lines 280-297)

Reviewer #2:

Comment 1

I only have on major concern and it is about the duration of treatment, did the authors take into consideration for how long these potential inappropriate psychotropic medicines were administered? Did the authors consider any minimum duration of treatment for each potential inappropriate psychotropic drug? Did the authors analyse if the risk of falls increased with the number of administered potentially inappropriate psychotropic medicines?

(1) I only have on major concern and it is about the duration of treatment, did the authors take into consideration for how long these potential inappropriate psychotropic medicines were administered?

Response:

Thank you for this comment. Yes, we have considered this point and added the following in the results section of the revised manuscript.
Exposure of residents to PIPMs:
"Supplementary Figure S1 displays the number of days residents were exposed to PIPMs and the proportion of days covered by PIPMs. Number of days residents were exposed to PIPMs represent the total number of days residents received a specific PIPM during their stay, while proportion of days covered by PIPMs is the ratio of number of days residents were exposed to PIPMs to the resident's length of stay. Proportion of days covered indicates the proportion of time residents were exposed to the PIPM during their stay in RACFs. Both metrics were calculated for overall PIPM usage as well as for different PIPM classes.
The overall median number of days residents were exposed to PIPMs were 91 days (IQR 6-320). The median number of days residents were exposed to PIPMs classes ranged from 11 days (IQR 2-160)

for benzodiazepines and Z drugs to 169 days (IQR 59-454) for antidepressants (Supplementary Figure SA). The median proportion of days covered by at least one PIPM was 39.3% (IQR 2.6-86.6%) indicating that half of the residents received PIPM for 2 out of 5 days. The median proportion of days covered across the PIPM classes ranged from 5.7% (IQR 0.8-62.6) for benzodiazepines and Z drugs to 63.1% (IQR 19.3-91.3) for antidepressants (Supplementary Figure SB). (Page 6 lines 166-177)

(2) Did the authors consider any minimum duration of treatment for each potential inappropriate psychotropic drug?

Response:

Thank you for this comment. Yes, we have considered this point and added the following in the result section of the revised manuscript.

Exposure of residents to PIPMs:

"Supplementary Figure S1 displays the number of days residents were exposed to PIPMs and the proportion of days covered by PIPMs. Number of days residents were exposed to PIPMs represent the total number of days residents received a specific PIPM during their stay, while proportion of days covered by PIPMs is the ratio of number of days residents were exposed to PIPMs to the resident's length of stay. Proportion of days covered indicates the proportion of time residents were exposed to the PIPM during their stay in RACFs. Both metrics were calculated for overall PIPM usage as well as for different PIPM classes.

The overall median number of days residents were exposed to PIPMs were 91 days (IQR 6-320). The median number of days residents were exposed to PIPMs classes ranged from 11 days (IQR 2-160) for benzodiazepines and Z drugs to 169 days (IQR 59-454) for antidepressants (Supplementary Figure SA). The median proportion of days covered by at least one PIPM was 39.3% (IQR 2.6-86.6%) indicating that half of the residents received PIPM for 2 out of 5 days. The median proportion of days covered across the PIPM classes ranged from 5.7% (IQR 0.8-62.6) for benzodiazepines and Z drugs to 63.1% (IQR 19.3-91.3) for antidepressants (Supplementary Figure SB). (Page 6 lines 166-177)

(3) Did the authors analyse if the risk of falls increased with the number of administered potentially inappropriate psychotropic medicines?

Response:

Thank you for this comment. We have considered proportion of days covered by each PIPM, but we did not cover a dose-response relationship because of less clinical information. We have added the following in limitation section (part of discussion) of the revised manuscript.

"Another limitation is that we could not find the dose response relationship to know if the risk of falls increased with the number of administered potentially inappropriate psychotropic medicines because of limited clinical information in our dataset". (page 14 lines 329-331)

VERSION 2 - REVIEW

**Reviewer**	**1**
**Name**	**Tan, Maw Pin**
**Affiliation**	**Faculty of Medicine, Department of Medicine**
**Date**	**21-Feb-2025**
**COI**	

The authors have addressed concerns that were raised during the first review, and made the necessary changes within their manuscript.

While its undeniable that the authors have indeed conducting an excellent enquiry and produced a wonderfully written paper packed with very useful information, I still have one more concern.

As clearly indicated by the authors, they only used the CNS section of the Beers criteria. Hence the label PIPM used throughout the manuscript is potentially misleading, since the use of non-CNS PIMs have not been considered.

There are some minor comments which I have included within the annotated pdf document attached.

The reviewer provided a marked copy with additional comments. Please contact the publisher for full details.

**Reviewer**	**2**
**Name**	**Agustí , Antonia**
**Affiliation**	**Hospital Universitari Vall d'Hebron, Clinical Pharmacology**
**Date**	**28-Feb-2025**
**COI**	

No more comments.

VERSION 2 - AUTHOR RESPONSE

Reviewer #1:
Dr. Maw Pin Tan, Faculty of Medicine
Comments to the Author:

Comment 1
The authors have addressed concerns that were raised during the first review and made the necessary changes within their manuscript. While its undeniable that the authors have indeed conducting an excellent enquiry and produced a wonderfully written paper packed with very useful information, I still have one more concern.

As clearly indicated by the authors, they only used the CNS section of the Beers criteria. Hence the label PIPM used throughout the manuscript is potentially misleading, since the use of non-CNS PIMs have not been considered.

Response:

Thank you for this comment and for highlighting this important consideration. In response to reviewer's feedback, we have revised the manuscript to replace the term "PIPM user" with "CNS-PIPM user" throughout.

Minor comments
There are some minor comments which I have included within the annotated pdf document

attached.

Comment 1

Using contemporaneous data on medications administered to residents, we aimed to 74 conduct a retrospective, dynamic and longitudinal cohort study. Am not sure the word dynamic is necessary.

Response:

Thank you for this comment. In response to the reviewer's feedback, we have removed the word 'dynamic' from our revised manuscript. (page 3 line 74)

Comment 2

Residents who were using at least one potentially inappropriate CNS medicine were classified as a PIPM user. while the authors have provided adequate explanation for only detecting CNS drugs, the use of the term PIPM user is then potentially misleading. The prevalence of CNS-PIPM use cannot be equated to all PIPM use since non-CNS PIPM would not have been detected.

Response:

Thank you for this comment and for highlighting this important consideration. In response to reviewer's feedback, we have revised the manuscript to replace the term "PIPM user" with "CNS-PIPM user" throughout.

Comment 3

The most frequently used PIPMs categories were benzodiazepines and Z-drugs 193 (27.4%), followed by antipsychotics first and second generation (17.2%) and antidepressants. this needs rephrasing

Response:

Thank you for your insightful comment. In response to the reviewer's feedback, we have rephrased 'antipsychotics first and second generation' for improved clarity in our revised manuscript. The revised text is presented below

"The most frequently used PIPMs categories were benzodiazepines and Z-drugs (27.4%), followed by first- and second-generation antipsychotics (17.2%) and antidepressants with strong anticholinergic activity (5.22%)." (page 7 line 193-194)

Comment 4

Another study among 4,502 residents 264 from 41 nursing homes in Spain found that residents on psychotropic medicines 265 experienced falls with 8.8% residents experienced at least 1 fall. The word experienced needs rephrasing.

Response:

Thank you for this comment. In response to the reviewer's feedback, we have rephrased the word 'experienced' in our revised manuscript. The revised text is presented below

"Another study among 4,502 residents from 41 nursing homes in Spain found that residents on psychotropic medicines experienced falls with 8.8% of them having at least 1 fall during one month study period [32]". (page 12 line 267)

Comment 5

Our findings have practical implication. this sentence is neither grammatically correct nor necessary.

Response:

Thank you for this comment. In response to the reviewer’s feedback, we have removed the sentence’ our findings have practical implication’ from our revised manuscript. (page 12 line 281)

Comment 6

According to recent guidelines on fall prevention and management among older adults, medicines should be reviewed at least once in a year and every 6 months among frail residents. this statement needs a little clarification to ensure the reader recognizes that once in a year applies to all older adults

Response:

Thank you for this comment. In response to the reviewer’s feedback, we have clarified this statement in our revised manuscript. The revised text is presented below

“According to recent guidelines on fall prevention and management among older adults, medicines should be reviewed at least once in a year for all older adults and every 6 months among frail residents”. (page 13 line 316-317)

Comment 7

This supports recommendations to regularly review psychotropic medicine use and ensure use is considered when developed falls prevention programs. should this be "developing"

Response:

Thank you for this comment. In response to the reviewer’s feedback, we have revised the wording in our manuscript, replacing "developed" with "developing”. (page 14 line 340)

Reviewer #2:

Dr. Antonia Agustí , Hospital Universitari Vall d'Hebron, Universitat Autonoma de Barcelona
Comments to the Author:
No more comments.

Response:

We thank the reviewer for their positive assessment.
